# Clinical Management of Intermittent Fasting in Patients with Diabetes Mellitus

**DOI:** 10.3390/nu11040873

**Published:** 2019-04-18

**Authors:** Martin M. Grajower, Benjamin D. Horne

**Affiliations:** 1 Division of Endocrinology, Albert Einstein College of Medicine, Bronx, NY 10463, USA; grajower@msn.com; 2 Director of Cardiovascular and Genetic Epidemiology, Intermountain Medical Center Heart Institute, and Department of Biomedical Informatics, University of Utah, Salt Lake City, UT 84107, USA

**Keywords:** intermittent energy restriction, intermittent fasting, alternate-day fasting, periodic fasting, time-restricted feeding

## Abstract

Intermittent fasting is increasing in popularity as a means of losing weight and controlling chronic illness. Patients with diabetes mellitus, both types 1 and 2, comprise about 10% of the population in the United States and would likely be attracted to follow one of the many methods of intermittent fasting. Studies on the safety and benefits of intermittent fasting with diabetes are very limited though, and health recommendations unfortunately today arise primarily from weight loss gurus and animal studies. Medical guidelines on how to manage therapeutic intermittent fasting in patients with diabetes are non-existent. The evidence to build such a clinical guideline for people with a diabetes diagnosis is almost non-existent, with just one randomized trial and several case reports. This article provides an overview of the available knowledge and a review of the very limited pertinent literature on the effects of intermittent fasting among people with diabetes. It also evaluates the known safety and efficacy issues surrounding treatments for diabetes in the fasting state. Based on those limited data and a knowledge of best practices, this paper proposes expert-based guidelines on how to manage a patient with either type 1 or 2 diabetes who is interested in intermittent fasting. The safety of each relevant pharmaceutical treatment during a fasting period is considered. When done under the supervision of the patient’s healthcare provider, and with appropriate personal glucose monitoring, intermittent fasting can be safely undertaken in patients with diabetes.

## 1. Introduction

The term intermittent fasting connotes reduced caloric intake on an intermittent basis. This can vary from several hours during the day to a complete 24-h period. It can be done for religious reasons, such as during Ramadan or Yom Kippur, or for health reasons, including weight loss. In this article, we will address only non-religious intermittent fasting conducted for health purposes and will review the benefits, either potential or proven, as well as safety concerns in patients with diabetes mellitus, both types 1 and 2. Articles have been written on how to manage religious fasting and the reader interested in this topic is referred to these articles [1,2,3,4].

## 2. Definition

The term intermittent fasting, when used for health reasons or weight loss, has been used to describe various types of caloric restriction (see Table 1). Some authors use it when a patient withholds caloric intake for several consecutive hours during the day (often 16 h with all energy intake during the other 8 h of the day) [5], others for a full day once or twice a week [6], and others three or four days per week [7]. Some protocols allow protein intake but no carbohydrates and still label it intermittent fasting [8]. Others allow carbohydrates or macro/micro-nutrients up to a limit that will still promote ketosis and, although it is simply a low-calorie diet, due to the popularity of fasting this has been labeled a diet that mimics fasting [9]. In all instances, non-caloric fluid intake is permitted (which is one of the main differences when compared to religious fasting) and therefore significantly reduces the risk of dehydration and hypotension, a prominent consideration in religious fasting.

## 3. Mechanism of Action

Most studies of intermittent fasting have focused on weight loss as the primary goal [7,8,10,11,12]. Those studies were conducted under the concept that the primary health benefit of intermittent fasting arises from weight loss. Because of this, the time-restricted feeding, alternate-day fasting, and 5:2 diet regimens are not intended to be ketotic, but to primarily induce improvements in health through the typical mechanisms associated with weight loss. For a more thorough review of human studies of the effect of intermittent fasting on changes in weight, we suggest a review paper by Malinowski and colleagues (and specifically Section 5 and Table 5 in Malinowski) [13].

While ketosis is neither a goal nor an expectation of those meal timing plans, some fasting regimens may achieve ketosis. Anton et.al. [14] have used the term “metabolic switch” to describe “the body’s preferential shift from utilization of glucose from glycogenolysis to fatty acids and fatty acid-derived ketones” (pg. 255). They point out that “ketones are the preferred fuel for both the brain and body during periods of fasting and extended exercise” (pg. 255) [14].

The metabolic switch occurs when glycogen stores in the liver are depleted, generally 12 h after the cessation of food intake, and adipose tissue lipolysis increases to produce more fatty acids and glycerol. The free fatty acids are transported to the liver where they are oxidized to β-hydroxybutyrate and acetoacetate. They are converted to energy through beta-oxidation. Generally, this process involves increased circulating fatty acids and other changes related to glucose and fatty acid metabolism, whose changes were recently reported among humans during water-only fasting [15].

Peroxisome proliferator-activated receptor alpha (PPAR-α) induces the expression of genes that mediate fatty acid oxidation in muscle cells. Interestingly, insulin resistance prolongs the time it takes to flip the metabolic switch and thus among people with diabetes it may take longer to begin using fatty acids for energy. All of the implications of this difference are not understood but potentially have implications for management of people with diabetes who engage in intermittent fasting [14], but this requires investigation in people with diabetes.

In those regimens that do not involve true fasting (see Table 1), the “metabolic switch” mechanism would not engage and presumably the mechanism of action is simply decreased caloric intake. Other potential mechanisms of health benefits from fasting are under study currently. These include the potential impact of intermittent fasting on inflammation, reactive oxygen species, blood pressure, and cholesterol levels [13,16], some of whose changes may occur simply due to weight loss but that may potentially also be impacted through mechanisms that are independent of weight change. They also may include an impact on the human microbiome [15,16], the human growth hormone/insulin-like growth factor-1 axis [16,17], mitochondriogenesis [16], immune system efficiency [16], and autophagy. [15,16] Autophagy regulates the amino acid supply, and this was recently reported to be controlled in specific patterns during water-only fasting in humans [15]. Previously, a pattern of increased oxygen carrying capacity through higher erythrocyte count and hemoglobin levels during water-only fasting was reported that may improve metabolic functioning or decrease insulin resistance [17]. Other mechanisms may also exist that are just beginning to be explored. Further evaluation of the mechanisms of possible health effects of intermittent fasting in humans is needed to fully understand the impact that it has on human health.

## 4. Benefits

Insulin resistance, the most prominent feature of type 2 diabetes, has long been known to improve with caloric restriction [18]. After a period of fasting, insulin sensitivity rises and insulin levels fall [11,12]. These result in improved fasting and postprandial glucose levels. In addition, as insulin induces adipose tissue growth, there is less propensity to weight gain and potentially even weight loss.

Intermittent fasting can thus be expected to influence weight loss, especially when it is conducted frequently. Early in the study of fasting’s health effects it was hypothesized that fasting could ameliorate some of the major undesirable effects of weight loss diets [10]. Intermittent fasting has now been shown, however, in various small and short-term studies to be similarly effective as daily calorie restriction in producing weight loss [7,8]. Thus, when done frequently enough, fasting can be one option for healthy weight loss, but the best evidence indicates that fasting is not a superior weight loss method [8,10].

Insulin resistance is associated with an increased inflammatory state including elevated C-reactive protein, decreased adiponectin, lower low-density lipoprotein (LDL) particle size, and other metabolic factors that all contribute to or are associated with atherosclerosis and development of coronary artery disease [19].

Furthermore, insulin is known to be both atherogenic as well as increase the risk of fluid retention and congestive heart failure [20,21]. Thus, reducing insulin levels through intermittent fasting would have the potential for reducing major adverse cardiovascular events. Such reduction in insulin may be achievable. Furmli et.al. [5] reported on three patients who were able to discontinue insulin treatment 5–18 days after beginning intermittent fasting, during which they ate dinner but skipped breakfast and lunch on either alternate days or 3 days per week. Further investigation of this hypothesis in larger populations is needed, but this finding is a tantalizing and potentially paradigm-shifting result if it can be safely and reliably repeated in large populations.

Intermittent fasting and calorie restriction have been shown to improve various metabolic and inflammatory pathways. Included are increased heat shock protein, promoting cellular autophagy, reducing advanced glycation end products, increased adiponectin, and decreased inflammation cytokines [22]. Each of these effects result in decreased vascular dysfunction and would therefore be expected to improve cardiovascular risk and/or mortality. Whether in fact the changes due to fasting are significant and sustained enough to do so remains to be proven.

While there are no prospective clinical trials of cardiovascular benefits from intermittent fasting (i.e., its effects on clinical major adverse cardiovascular events), observational population studies have shown cardiovascular and metabolic benefits—a lower risk of coronary artery disease and lower risk of diabetes—from as little as one day per month of energy restriction through fasting (practiced over a period of decades) [23]. One prospective clinical trial did recently report an effect of intermittent fasting on the control of hemoglobin A_1c_ [8]. Among a population of 97 people with type 2 diabetes mellitus (40 of the 137 enrolled in the trial withdrew early), hemoglobin A_1c_ reduction due to intermittent fasting was non-inferior to continuous energy restriction [8]. Unfortunately, weight loss in that trial was not different in the fasting arm compared to caloric restriction, and other metabolic measures were not different [8]. Overall, reviews of the evidence show that insufficient human data exist presently to recommend the use of intermittent fasting or low-calorie diets to prevent diabetes or, among people with diabetes, to prevent its sequelae [24,25].

## 5. Risks

The most immediate risk with intermittent fasting is the potential for hypoglycemia in patients who are on antidiabetic medications that are associated with hypoglycemia, specifically insulin (both prandial and basal) and sulfonylureas (including the short-acting meglitinides) [6,8,26]. All other antidiabetic medications when used either as monotherapy or in combination therapy without insulin or sulfonylureas are rarely, though not never, associated with hypoglycemia, and the risk is therefore considerably less though still a consideration.

With long-term intermittent fasting, one needs to also be concerned about protein malnutrition if patients are not cognizant to maintain adequate protein intake on those days when they are eating. Vitamin and mineral malnutrition could also occur and, depending on how many days a week the patient is fasting and what they are eating on the days they do eat, might necessitate taking vitamin and/or mineral supplements.

Other risks include a variety of potential harms related to insufficient energy intake and some due to dehydration. These include safety events that may occur among anyone who engages in intermittent fasting, regardless of whether they have diabetes. Such adverse events may include dizziness, nausea, insomnia, syncope, falls, migraine headache, weakness that limits daily activities, and excessive hunger pangs. The presence of a chronic disease, including diabetes, may increase the risk of experiencing many of these adverse events, as may other diseases including coronary artery disease, unstable angina, heart failure, atrial fibrillation, prior myocardial infarction, prior stroke or transient ischemic attack, most cancers, chronic obstructive pulmonary disease, pulmonary embolism, asthma, peripheral vascular thromboembolism, chronic kidney disease, and potentially other conditions. For people with these chronic diseases, little is known about the response to fasting, thus it is not necessarily that they should not engage in fasting, but that how their risks due to fasting are changed is uncertain and require studies to be conducted in these populations where elevated health risks exist. Certainly, exposing such individuals to serious adverse events such as new myocardial infarction, stroke, or death is unwarranted and caution is the key presently given the lack of evidence in these populations.

For conditions where dehydration is a risk, such as stroke [27,28], encouragement to hydrate well during any fasting regimen is recommended. Drinking water, including to replace fluids that normally would be consumed in foods, is an important consideration for people of all ages who are participating in intermittent fasting.

Furthermore, some populations have unique risks and should be dissuaded from engaging in intermittent fasting, especially if they have diabetes. This includes pregnant and lactating women, young children, adults of advanced age, and older adults who are frail. Individuals with immunodeficiencies, including those who have had a solid organ transplant and are on medical immunosuppression, should also refrain from fasting. People with eating disorders and those with dementia have unique challenges that will likely be exacerbated by deliberately engaging in fasting, thus they should not follow intermittent fasting regimens. Patients who have a history of traumatic brain injury or post-concussive syndrome may also be at higher risk of adverse events, and their needs should be considered carefully on a case-by-case basis prior to beginning a fasting regimen.

## 6. Management

Patients with diabetes who are interested in intermittent fasting should be encouraged to engage in fasting with guidance from a healthcare practitioner, including physicians, nurse practitioners, physicians’ assistants, certified diabetes educators, or registered dietitians. Specific attention should be paid to three considerations: medication adjustment, frequency of glucose monitoring, and fluid intake [26]. Most of these recommendations are based on the clinical experience of the authors when there is no available literature, while some of the recommendations are based on published approaches in studies of intermittent fasting among people with diabetes [6,8].

### 6.1. Medication Adjustment

Anti-diabetes medications, including sulfonylureas, meglitinides, and insulin, are associated with hypoglycemia and their doses should be adjusted on days of intermittent fasting.

The adjustment should take into consideration the control of the patient’s diabetes including both fasting and postprandial glucose levels over the preceding 2–4 weeks, short-acting (prandial insulin or insulin pump, nateglinide and repaglinide) or long-acting (basal insulin, glyburide, glipizide, glimepiride) therapy, the duration of the fasting, and whether the fasting precludes all caloric intake or just carbohydrates. Specific recommendations for each of these medications and other classes of agents are provided below in Table 2.

#### 6.1.1. Metformin, Thiazolidinedione (TZD), Dipeptidyl Peptidase 4 (DPP-4) Inhibitors

Metformin, thiazolidinedione’s (pioglitazone and rosiglitazone), and DPP-4 inhibitors rarely cause hypoglycemia and these medications can be continued as usual. Metformin and TZDs have beneficial effects other than just glucose control, and they therefore should be continued. DPP4 inhibitors, on the other hand, are prescribed just for glucose control, so if the patient or provider prefers they could be skipped on the day of fasting.

#### 6.1.2. Sodium-Glucose Cotransporter 2 (SGLT-2) Inhibitors

SGLT-2 Inhibitors also rarely cause hypoglycemia; however, they do also cause osmotic diuresis. Because of this latter effect, if there are changes in the patient’s usual fluid intake during the fasting day, it might be appropriate to skip taking this medication on days on which the patient engages in intermittent fasting to avoid dehydration and the resultant hypotension.

#### 6.1.3. Sulfonylureas

Glyburide, glimepiride, and glipizide are long-acting sulfonylureas and are often associated with hypoglycemia during reduced caloric intake [26]. These medications should always have their doses reduced on the date of fasting to avoid potential adverse events. Further, if the patient takes these medications in the evening, the dose taken on the evening prior to the date of fasting should also be reduced or withheld for safety purposes.

#### 6.1.4. Meglitinides

Nateglinide and repaglinide are short-term sulfonylureas. The incidence of hypoglycemia from these medications is less than with sulfonylureas [26]. As these pharmaceutical agents are generally prescribed for the control of postprandial hypoglycemia, they should be withheld prior to any meal that the patient will be eating that contains no carbohydrates.

#### 6.1.5. Glucagon-Like Peptide-1 (GLP-1) Receptor Analogues

GLP-1 receptor analogues reduce hyperglycemia in a glucose-dependent manner [29] and are therefore rarely associated with hypoglycemia. Certainly, for the once-weekly dulaglutide, albiglutide, semaglutide, and exenatide-LA, a dose adjustment is not possible. For the daily liraglutide, however, while withholding the drug on the day of fasting is feasible, the risk of hypoglycemia is quite low. Because withholding the daily liraglutide dose could adversely affect the next morning’s fasting glucose, it therefore should be continued in spite of an intermittent fasting regimen. In contrast, once-daily lixisenatide only lowers postprandial glucose and therefore should be withheld on the day of fasting as there would not seem to be a benefit in taking it. There are no evidence-based studies to support these recommendations though, so provider evaluation of the patient and his or her individual needs and intermittent fasting regimen are suggested.

#### 6.1.6. Alpha Glucosidase Inhibitors and Bile Acid Sequestrant

The alpha glucosidase inhibitors acarbose and miglitol, as well as the bile acid sequestrant colesevelam, inhibit the absorption of carbohydrates [30]. These medications are given pre-prandially for optimal effect. Given this dosing approach, they should be withheld prior to any meal that is being skipped or that does not contain any carbohydrates. This is primarily because there would be no benefit in taking them during a fasting period, not because of a risk of hypoglycemia. In the case of colesevelam, however, if it is being given also for the purpose of reducing cholesterol, it should be continued on days of intermittent fasting.

#### 6.1.7. Dopamine Agonist

Bromocriptine acts through an uncertain mechanism but appears to affect the sympathetic output of glucose [31]. The risk of hypoglycemia is low with this pharmaceutical agent and it can be continued throughout intermittent fasting.

#### 6.1.8. Basal Insulin

Adjustments in basal insulin in conjunction with intermittent fasting need to take into account the patient’s fasting blood sugar as well as the risk of hypoglycemia, including hypoglycemia unawareness. In addition, the duration of action of the basal insulin needs to be factored into the considerations for each patient.

In a patient starting out with fasting blood sugars that are considered controlled for that patient, the patient may be more susceptible to hypoglycemia during the day of fasting or the following morning. If the patient has hypoglycemia unawareness or is at increased risk for hypoglycemia complications, the dose of basal insulin should be decreased and the patient should be encouraged to do more frequent blood glucose testing until a stable pattern is demonstrated based on the type of intermittent fasting (see Table 1). 

The dose of glargine 1% (Lantus or Basaglar), detemir (Levemir), or neutral protamine hagedorn (NPH, or isophane) should initially be reduced by one half in the patient considered well-controlled, to one third in a patient not well-controlled. In contrast, degludec (Tresiba) and glargine 3% (Toujeo) have a 36–42 h half-life; reducing the dose on the day of fasting will therefore not be expected to have any effect on that day but on the subsequent day. Accordingly, these insulins should either not be adjusted at all, or the dose may need to be adjusted on a daily basis during the duration of the intermittent fasting in order to accommodate the intermittent fasting days in order to avoid hypoglycemia. Here too, the judgment of the treating practitioner must take into account the degree of the patient’s control prior to starting the fasting and the risk for hypoglycemia. When in doubt about how much to reduce the dose, it is advisable to err on the side of caution in reducing the dose of insulin, and then to adjust the dose up (or down) based on more frequent blood glucose testing as a fasting regimen proceeds.

Since these are not religious fasts wherein the patient may be reluctant to “break the fast” in the case of dropping blood sugars, a patient beginning intermittent fasting while on insulin should be given specific guidelines on when and what to eat if the blood glucose drops below a specific number (individualized for that patient) with subsequent adjustment in the basal insulin going forward. The patient should have the concept reinforced that the potential long-term health benefits of fasting are always outweighed by the short-term risks due to hypoglycemia.

Patients on an insulin pump should initially reduce their basal rate by 10%, with further adjustments based on frequent (every two hours) blood glucose testing that continues until a stable pattern is established. It is not unusual with a full day fast to encounter a need for the basal rate to be reduced by as much as 90% towards the end of the fasting day in a patient with type 1 diabetes. The pattern of measurement and repeated adjustment should be followed throughout the fast to ensure the safety of the patient.

#### 6.1.9. Prandial Insulin

Prandial, or mealtime, insulin (including regular, lispro, glulisine, and aspart), whether used as part of a multiple daily injection regimen or an insulin pump, should not be taken if a meal is going to be completely skipped. If the patient will be consuming some food during a fasting period, an appropriate reduction in insulin based on any carbohydrates consumed would be indicated.

#### 6.1.10. Amylinomimetics

Symlin (pramlintide) reduces postprandial hypoglycemia by reducing meal-associated glucagon secretion [32]. It is injected with prandial insulin prior to meals. Because of this, its use should therefore be withheld if the patient will also not be injecting their prandial insulin.

### 6.2. Glucose Monitoring

Unless the patient is using a sulfonylurea or insulin, the risk of hypoglycemia is low and no additional glucose monitoring would be routinely recommended during fasting. The patient should, however, be reminded about the symptoms of hypoglycemia and should be encouraged to check their blood glucose if any of the symptoms do develop. Some patients will develop symptoms suggestive of hypoglycemia even with a blood glucose above 70 mg/dL, thus caution is indicated in the approach to their response to symptoms in conjunction with glucose monitoring [33].

In patients on a sulfonylurea or insulin (either alone or in combination with any other antidiabetic medication), the risk of hypoglycemia is significant, and the patient should be encouraged to do more frequent blood glucose testing, especially when first starting out with intermittent fasting. Depending on the risk of hypoglycemia as assessed by the practitioner, the testing could be as often as every two hours in the patient on insulin or every four hours on sulfonylureas. If the fasting is a 24-h or longer fast, especially a water-only fast, specific attention to the next morning’s fasting blood glucose reading should be made.

Patients on insulin who are going to undertake intermittent fasting might be encouraged to use personal continuous glucose monitoring systems. In the case of the Dexcom system, this would allow for a hypoglycemia alert. With the Abbott Freestyle Libre system, while there is no hypoglycemia alert, frequent testing could be done without additional expense or discomfort. The risk of hypoglycemia with intermittent fasting while using insulin cannot be over emphasized and may even increase if the patient is successful in losing weight as a result of the intermittent fast. While relying on fingerstick glucose testing may be adequate, having a continuous glucose monitoring system would generally encourage the patient to do more frequent glucose testing and afford the additional safety that comes with more frequent testing.

### 6.3. Fluid Intake

While patients will be drinking non-caloric liquids during intermittent fasting, patients may not realize that unless they drink additional liquids, they are actually reducing their total fluid intake due to reduced intake of foods such as soups, yogurt, or melons. In this case, the risk of dehydration and hypotension increases. The patient may then need to reduce or withhold their intake of diuretics, SGLT-2 inhibitors, or anti-hypertensive medications on the days of fasting. 

## 7. Conclusions

Intermittent fasting, when undertaken for health reasons in patients with diabetes mellitus, both types 1 and 2, has been shown in a few small human studies to induce weight loss and reduce insulin requirements. While these findings are exciting and have captured the imagination of many people, a wise approach to implementing fasting regimens and using them in the long term among this specific population is required. Much of the hype surrounding fasting arises from animal studies, which only suggest what human research should be conducted; implementation of human interventions should not be based on animal research.

Long-term benefits of fasting, including cardiovascular risk reduction, remain to be fully studied and elucidated, especially in humans. Clinicians should temper the enthusiasm for fasting with the reality that the benefits and risks in humans remain largely unexplored and the benefits may take months to years to appear or be fully realized. Good evidence from epidemiologic studies, pilot interventional trials, and a few randomized trials does suggest that the benefits of fasting outweigh the potential harms in the average individual. People with diabetes, however, are not the average individual, and their personal needs require more careful consideration at the beginning of and during the use of a fasting regimen. With proper medication adjustment and self-monitoring of blood glucose levels though, intermittent fasting can be encouraged and safely implemented among people with diabetes.

## Figures and Tables

**Table 1:** Different protocols labeled intermittent fasting.

Protocol	Frequency	Duration	Additional Considerations
Time-Restricted Feeding	Every day	16 h	Feeding occurs during the day’s other 8 h, usually early in the day after rising from bed. A more restrictive variant limits feeding to 6 h during the day and fasting occurs for 18 h.
Alternate-Day Fasting	Every other day	24 h	One ≈ 500 calorie meal * is consumed at about the mid-point or ≈ 12 h into a 24-h period. For example, in one study, subjects were “instructed to consume 25% of baseline energy intake as a lunch (between 12 pm and 2 pm) on fast days…” (pg. 931) [10]. When a meal is included, technically this is a non-fasting very-low-calorie regimen or “partial fast.”
“5:2 Diet”	Twice per week	24 h	One 500–600 calorie meal * is consumed on the fasting day. For example, one study instructed subjects to follow “a diet of 500 to 600 kcal/day for 2 days of the week…” (pg. 3) and most fasting days were non-consecutive [8]. When a meal is included, technically this is a non-fasting very-low-calorie regimen, or “partial fast”.
Weekly One-Day Fasting	Once per week	24 h	A water-only fasting regimen.
Fast-Mimicking Diet	Once per month	120 h	A low-calorie non-fasting ketogenic diet. This is a non-fasting regimen allowing small maximum amounts of macronutrients.
Ten-day Juice Fast	Irregular frequency	240 h	Fruit juices or broths are consumed during the fasting period, but no solid foods.
Other Regimens	Varied	Varied	Many possible frequency- and timing-based approaches are possible.

* The meal may be optional and its timing during the fasting day may vary, depending on the specific regimen that is being followed.

**Table 2:** Considerations and recommendations for adjustment of antidiabetic medications during intermittent fasting.

Class of Medication	Drugs	Risk of Hypoglycemia	Dose Adjustment	Comments
Biguanides	metformin	low	None	
Thiazolidinediones	pioglitazone, rosiglitazone	low	None	
Sulfonylureas	glyburide, glipizide, glimepiride	high	Skip that day for a 24-h fast; as utilized in one study [6], take half the dose for a partial day fast (i.e., when a meal is consumed at some point part way through the fasting day)	A caution for the half dose is that substantial education and monitoring may be required to avoid hypoglycemia [6]. Another study skipped the whole dose on any even partial fasting day, which is more conservative and cautious [8].
Meglitinides	nateglinide, repaglinide	moderate	Skip prior to a meal containing no carbohydrates	
DPP4 Inhibitors	saxagliptin, sitagliptin, alogliptin, linagliptin	low	None (or can skip on the day of fasting)	The dose can be skipped because there is no benefit to taking it and this would reduce healthcare costs to the patient.
SGLT2 Inhibitors	dapagliflozin, empagliflozin, canagliflozin, ertugliflozin	low	Can skip on the day of a 24 h fast OR should skip if concern for dehydration exists	The dose can be skipped because there is no benefit to taking it and this would reduce healthcare costs to the patient.
GLP-1 Receptor Analogues, weekly	dulaglutide, albiglutide, semaglutide, exenatide-XR	low	None	
GLP-1 Receptor Analogues, daily	liraglutide, lixisenatide	low	None	For lixisenatide only, with a 24-h fast, can skip the dose
Alpha glucosidase inhibitors	acarbose, miglitol	low	Skip if patient not eating carbohydrates that meal	
Bile Acid Sequestrants	colesevelam	low	Skip	If the primary indication is for lowering cholesterol, dose should be taken
Dopamine Agonists	bromocriptine	low	None	
Basal Insulin (note: one study decreased basal insulin by 50% on fasting days and still had significant hypoglycemia rates [6], thus caution is required)	NPH, Levemir, glargine 1%, Basaglar	high	Take one-third of usual dose (67% lower dose) for controlled patient; take half of usual dose (50% lower dose) for uncontrolled patient	Definition of controlled and uncontrolled at the discretion of the treating physician based on risk for hypoglycemia. Monitor closely and proactively.
	glargine 3%, degludec	moderate	None initially	Monitor closely and proactively; reduce dose if fasting glucose goes below a pre-specified number
Prandial insulin (note: one study decreased prandial insulin by 70% on fasting days and still had significant hypoglycemia rates [6], thus caution is required)	lispro, aspart, glulisine	high	Skip dose if patient not eating carbohydrates at that meal	Monitor closely and proactively
Insulin Pump		high	Adjust basal rate starting at 10% and reducing further based on glucose monitoring; Adjust bolus based on carbohydrate intake at next meal	Monitor closely and proactively
Combination insulins	70/30, 75/25, 50/50	high	Skip dose based on above guidelines for prandial insulin	
amylinomimetics	pramlintide	low		Take if patient is taking prandial insulin
